# Anterior cingulate gamma‐aminobutyric acid concentrations and electroconvulsive therapy

**DOI:** 10.1002/brb3.1833

**Published:** 2020-09-17

**Authors:** Vera J. Erchinger, Jeremy Miller, Thomas Jones, Ute Kessler, Juan Bustillo, Jan Haavik, Jonathan Petrillo, Gregory Ziomek, Åsa Hammar, Ketil J. Oedegaard, Vince D. Calhoun, Shawn M. McClintock, Lars Ersland, Leif Oltedal, Christopher C. Abbott

**Affiliations:** ^1^ Department of Clinical Medicine University of Bergen Bergen Norway; ^2^ Department of Psychiatry University of New Mexico School of Medicine Albuquerque NM USA; ^3^ Division of Psychiatry Haukeland University Hospital Bergen Norway; ^4^ Department of Biomedicine University of Bergen Bergen Norway; ^5^ Department of Psychiatry University of Texas at Austin Dell Medical School Austin TX USA; ^6^ Department of Biological and Medical Psychology University of Bergen Bergen Norway; ^7^ Tri‐institutional Center for Translational Research in Neuroimaging and Data Science (TReNDS) Georgia Tech Emory Atlanta GA USA; ^8^ Division of Psychology Department of Psychiatry University of Texas Southwestern Medical Center Dallas TX USA; ^9^ Division of Brain Stimulation and Neurophysiology Department of Psychiatry and Behavioral Sciences Duke University School of Medicine Durham NC USA; ^10^ Department of Clinical Engineering Haukeland University Hospital Bergen Norway; ^11^ Mohn Medical Imaging and Visualization Centre Department of Radiology Haukeland University Hospital Bergen Norway

**Keywords:** depression, electroconvulsive therapy, gamma‐aminobutyric acid, magnetic resonance spectroscopy

## Abstract

**Objective:**

The anticonvulsant hypothesis posits that ECT’s mechanism of action is related to enhancement of endogenous anticonvulsant brain mechanisms. Results of prior studies investigating the role of the inhibitory neurotransmitter gamma‐aminobutyric acid (“GABA+”, GABA and coedited macromolecules) in the pathophysiology and treatment of depression remain inconclusive. The aim of our study was to investigate treatment‐responsive changes of GABA+ in subjects with a depressive episode receiving electroconvulsive therapy (ECT).

**Methods:**

In total, 41 depressed subjects (DEP) and 35 healthy controls (HC) were recruited at two independent sites in Norway and the USA. MEGA‐PRESS was used for investigation of GABA+ in the anterior cingulate cortex. We assessed longitudinal and cross‐sectional differences between DEP and HC, as well as the relationship between GABA+ change and change in depression severity and number of ECTs. We also assessed longitudinal differences in cognitive performance and GABA+ levels.

**Results:**

Depressive episode did not show a difference in GABA+ relative to HC (*t*_71_ = −0.36, *p* = .72) or in longitudinal analysis (*t*_36_ = 0.97, *p* = .34). Remitters and nonremitters did not show longitudinal (*t*_36_ = 1.12, *p* = .27) or cross‐sectional differences in GABA+. GABA+ levels were not related to changes in antidepressant response (*t*_35_ = 1.12, *p* = .27) or treatment number (*t*_36_ = 0.05, *p* = .96). An association between cognitive performance and GABA+ levels was found in DEP that completed cognitive effortful testing (*t*_18_ = 2.4, *p* = .03).

**Conclusion:**

Our results failed to support GABA as a marker for depression and abnormal mood state and provide no support for the anticonvulsant hypothesis of ECT. ECT‐induced change in GABA concentrations may be related to change in cognitive function.

## Graphical abstract

In depressed subjects treated with ECT (*n* = 40), gamma‐aminobutyric acid (GABA+) levels did not change over treatment and we found no association between GABA+ levels and antidepressant response. However, change of GABA+ was associated with cognitive performance in a subset of patients (*n* = 24) who completed cognitive testing.

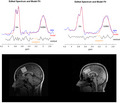

## SIGNIFICANT OUTCOMES

1. Gamma‐aminobutyric acid (GABA+) levels were not related to ECT antidepressant response.
2. Change of GABA+ was related to cognitive performance in subjects who completed cognitive testing.

## LIMITATIONS

1. As some of the subjects were using medication at the same time as they underwent ECT, an effect on GABA+ from these medications cannot be excluded.
2. Even though statistical analysis corrected for site, the sites used different types of anesthesia and different methods for calculation of ECT dosage, which could influence results.
3. The technologic limitations of magnetic resonance spectroscopy in GABA+ measurement limit the ability to detect shifts of GABA+ from intracellular to extracellular spaces (synaptic cleft).

## 1 INTRODUCTION

Electroconvulsive therapy (ECT) is efficacious for treatment‐resistant depression ( 2003). While the procedure has been in use for more than eight decades, the mechanism of action remains unknown. The anticonvulsant hypothesis of ECT provides a unique treatment‐specific theoretical framework for the antidepressant mechanism of action (Sackeim, 1999). This framework posits that the therapeutic outcome is related to enhancement of endogenous anticonvulsant brain mechanisms. Data that support this hypothesis include the observation that seizure threshold increases in some patients as they progress through the ECT series (Coffey, Lucke, Weiner, Krystal, & Aque, 1995; Krystal, Coffey, Weiner, & Holsinger, 1998; Sackeim, 1999). In addition, changes are noted in EEG seizure metrics including seizure morphology (ictal power, delta coherence, and postictal suppression) (Coffey et al., 1995; Perera et al., 2004) and increased electroencephalographic slow‐wave activity in the prefrontal cortex (Sackeim et al., 1996). Positron emission tomography has demonstrated decreased cerebral blood flow after ECT, which may be consistent with a hypometabolic state (Nobler et al., 1994). The hypometabolic state that occurs after ECT may be related to increased concentrations of gamma‐Aminobutyric acid (GABA), the predominant inhibitory neurotransmitter (Sackeim, Decina, Prohovnik, Malitz, & Resor, 1983).

Proton‐spectroscopy (1H‐MRS) measures chemical metabolites in the brain including glutamate and GABA. In standard 1H‐MRS of GABA, the signal also includes additional macromolecules; hence, the measurement is commonly denoted GABA+. Multiple investigations utilizing 1H‐MRS in depressed subjects have found inconsistent results. Two studies comparing GABA levels in medication‐free recovered depressed subjects with controls found low GABA concentrations in the occipital cortex, and the prefrontal (both dorsomedial/dorsal anterolateral and ventromedial prefrontal) and anterior cingulate cortex (ACC) (Bhagwagar et al., 2008; Hasler et al., 2007). Brennan et al. (2017) used 1H‐MRS to scan subjects with major depressive disorder (MDD) treated with citalopram. They found that greater increases in GABA+ in the pregenual anterior cingulate cortex from baseline to days 3 and 7 of citalopram treatment were significantly associated with clinical response. These small, positive studies provide some evidence that low cortical GABA+ could be a marker of a trait vulnerability to a mood disorder and a neurochemical correlate of an abnormal mood state. However, a larger study by Godlewska et al. investigated GABA+ in the occipital lobe using 1H‐MRS in depressed subjects treated with escitalopram and found no difference between subjects and healthy controls at baseline or changes in GABA+ during treatment (Godlewska, Near, & Cowen, 2015). Similarly, Hasler et al. (2005) examined GABA+ in the ventromedial prefrontal cortex and the dorsolateral/anterior medial prefrontal cortex and found no difference between subjects with MDD and healthy controls. Further complicating the picture is the significant heterogeneity in the depressed population. Price et al. used 1H‐MRS and found decreased GABA+ in the occipital lobe in subjects with treatment‐resistant depression versus subjects with nontreatment‐resistant depression and healthy controls (Price et al., 2009), while Gabbay et al. found decreased GABA+ in the ACC in young subjects who were stratified based on the presence of anhedonia only in the anhedonic MDD subgroup (Gabbay et al., 2017).

There are only two prior pre‐/post‐ECT 1H‐MRS investigations, which have also shown mixed results. In 2003, Sanacora et al. (2003) assessed GABA+ with 1H‐MRS in depressed subjects treated with ECT. Eight subjects with a diagnosis of MDD and a two‐week medication washout period received ECT. Pre‐/post‐ECT 1H‐MRS found a significant change in the mean GABA+ concentration in the occipital cortex, increasing from 0.85 mmol/kg brain tissue (*SD* = 0.34) to 1.51 (*SD* = 0.48) as seven of the eight subjects showed an increase. There was a concomitant decrease in depression severity, although no significant correlation was found between clinical response and change in GABA+. In 2018, Knudson et al. assessed pre‐/post‐ECT changes in the occipital and prefrontal cortices (Knudsen, Near, Blicher, Videbech, & Blicher, 2019). The inclusion of the prefrontal cortex, which has a role in regulation of emotional processing, was more relevant to depression‐related neurocircuitry (Kaiser, Andrews‐Hanna, Wager, & Pizzagalli, 2015). Eleven subjects with unipolar and bipolar depression were enrolled, and medications were continued throughout the study. They found no changes of GABA+ in the PFC, mean 0.249 (±0.04) versus 0.251 (±0.04), and no difference in the OCC (data not reported). Both studies acknowledged the limits of interpreting their results given the small sample sizes. In summary, 1H‐MRS investigations in depressed subjects focused on detection of treatment‐related changes of GABA+ have to date been inconclusive.

### 1.1 Aims of the study

We measured GABA+ in the ACC during a longitudinal investigation of ECT at two independent sites investigated the hypotheses that (a) ECT will be associated with increased GABA+ in the anterior cingulate in depressed subjects receiving ECT; and (b) changes in GABA+ will be related to clinical efficacy. An exploratory aim of this investigation was the assessment of GABA+ related to ECT‐mediated changes in effortful cognitive functioning.

## 2 MATERIAL AND METHODS

This pre‐/post‐ECT imaging investigation study was performed at the University of New Mexico Hospital and Haukeland University Hospital. Approval was obtained from both the Regional Committee for Medical and Health Research Ethics in Norway and the University of New Mexico Human Research Protections Office. For both study sites, the treatment group included depressed subjects with a severe depressive episode (DEP) who met criteria for treatment with ECT and were maintained on their psychotropic medications during the ECT series (see Table 2). Age (years) and gender‐matched healthy comparisons (HC) were concurrently recruited from the same geographic areas.

### 2.1 Inclusion and exclusion criteria

The study protocol at Haukeland University Hospital (HUH) has been previously reported (Oltedal et al., 2015). Inclusion criteria consisted of the following: unipolar or bipolar depressive episode, Montgomery‐Asberg Depression Rating Scale (MADRS) (Williams & Kobak, 2008) >25, clinical indication for ECT, and age >18 years. Exclusionary criteria included the following: ECT during the preceding 12 months, pregnant, inability to give written informed consent, or contraindications to MRI. HC had no psychiatric or neurological diagnoses and no medication use, except contraceptives. Twenty‐one DEP (48% female, mean age 44, *SD* ± 16 years) and 15 HC (60% female, mean age 41, *SD* ± 17 years) were included for joint analysis of GABA+ levels. Scans were conducted at four to six‐week time intervals for DEP (mean 38 days, range: 15–71 days) and HC (mean 40 days, range: 29–57 days).

At the University of New Mexico Hospital (UNMH), inclusion criteria consisted of the following: unipolar depressive episode and age range between 50 and 80 years. Additional inclusion and exclusionary criteria were identical to the Norway site. Twenty DEP (60% female, mean age 63, *SD* ± 10 years) and 20 HC (60% female, mean age 59, *SD* ± 6 years) were included for joint analysis of GABA+ levels. Scans were conducted at four to six‐week time intervals for DEP (42 days, range: 14–63 days) and HC (42 days, range: 28–53 days).

### 2.2 Assessments

MADRS (HUH) and 17‐item Hamilton Rating Scale for Depression (HDRS‐17; UNMH) confirmed depression severity. For combined analysis, the HDRS‐17 total score was converted to the MADRS total score (Heo, Murphy, & Meyers, 2007). Patients were classified as responders or remitters using the following criteria: ECT response was defined as more than 50% reduction in baseline MADRS and remission was defined as more than 50% reduction in baseline MADRS and a post‐ECT MADRS total score ≤ 10. Prescribed drugs during treatment and the number of ECT sessions administered were recorded.

Several neuropsychological tests were performed at both study site, whereas the Trail Making Test (Trails) Parts A and B (Reitan, 1958) was performed at both sites. Trails Part A measures simple visual scanning/attention, processing speed, number sequencing, which require low cognitive effort. Trails Part B measures complex visual scanning/attention, letter and number sequencing, processing speed, and cognitive flexibility, requiring high cognitive effort (Bowie & Harvey, 2006; Tombaugh, 2004). Although the Trails manual tends to cap the time for Part B at 300 s, we followed a testing of limits paradigm and allowed the participants as much as needed to complete the test (Lindenberger & Baltes, 1995). The raw scores (total time to test completion in seconds) were used for the analyses, and the change in scores between pre‐ and post‐testing was calculated.

### 2.3 ECT procedure and anesthesia

Thymatron System IV (Somatics LLC) was used at both sites. DEP were hyper‐oxygenated directly before and during the initiation of anesthesia to optimize induction of seizures. ECT parameters (electrode placement, amplitude, pulse width, frequency, duration, and total charge), anesthetics, and seizure duration were recorded for each treatment.

At HUH, DEP received right unilateral electrode placement (RUL) and 0.5 millisecond (ms) pulse width. Stimulus energy was determined by an age‐based method: Patient's age in years × 5 ≅ stimulus charge in mC (Abrams, (1988)). Anesthetic medications included thiopental (induction) and succinylcholine (neuromuscular blockade).

At UNMH, DEP started with RUL and 0.3 ms pulse width and received as needed bitemporal electrode placement (BT, 1.0 ms pulse width) in the context of RUL non‐response (*n* = 8). Seizure threshold was obtained during the first session with subsequent stimulus dosage at 6 × threshold for RUL and 2 × threshold for BT. Further adjustments to energy occurred as needed for inadequate seizure induction, defined as <25 s of electroencephalogram (EEG) seizure activity or poor seizure morphology. Anesthetic medications included methohexital (induction) and succinylcholine (neuromuscular blockade).

### 2.4 Structural and 1H‐MRS data acquisition

At HUH, a 3 Tesla GE Discovery 750 scanner system (Milwaukee, WI) with a 32‐channel head coil was used. For structural MRI acquisition, a 3D T1 weighted fast spoiled gradient echo (FSPGR) acquisition included the following parameters: Echo time/repetition time (TE/TR) = 2.9/6.7 ms, inversion time (TI) = 600 ms, flip angle = 8°, field of view (FOV) = 25.6 cm, and matrix size 256 × 256, giving isotropic voxel size of 1 × 1 × 1 mm. The FSPGR acquisition was used as localizer for positioning of the spectroscopy voxel across the midline in the anterior cingulate (ACC), angled in the sagittal plane aligned to the foremost slope of the corpus callosum (CC). For spectroscopic acquisition, 1H‐MRS single‐voxel point resolved spectroscopy (MEGA‐PRESS) (Mescher, Merkle, Kirsch, Garwood, & Gruetter, 1998; Mullins et al., 2014) included the following parameters: TE = 68 ms, TR = 1,500 ms, 384 averages in total, resulting in a total acquisition time of 10 min. The 1H‐MRS voxel measured 30 × 30 × 30 mm (27 ml).

At UNMH, a 3‐Tesla Siemens TIM Trio scanner (Malvern, PA) with a 32‐channel head coil acquired the imaging data. For structural MRI acquisition, a T1‐weighted multi‐echo magnetization‐prepared rapid acquisition with gradient echo (MPRAGE) included the following parameters: TR = 2.53 s; TE = 1.64, 3.5, 5.32, 7.22, 9.08 ms; TI = 1.2 s; flip angle = 7°; number of excitations = 1; slice thickness = 1 mm; FOV = 256 mm; matrix 256 × 256; and voxel size = 1 × 1 × 1 mm. For spectroscopic acquisition, the ACC location was similar to HUH and the acquisition parameters included the following: TE = 68 ms; TR = 2,140 ms; 256 averages in total, giving an acquisition time of 10 min. The 1H‐MRS voxel measured 40 × 26 × 20 (20.8 ml). Acquisition parameters included the following for HUH: spectral width was 5,000 Hz, 4,096 datapoints, with the ON editing pulse placed at 1.83 ppm and the OFF editing pulse placed at 7.43 ppm using a 16 ms sinc‐weighted Gaussian edit pulse. The water peak is assumed to be at 4.68 ppm. Acquisition parameters included the following for UNMH: spectral width was 1,200 Hz, 2,048 datapoints, with the ON editing pulse placed at 1.90 ppm and the OFF editing pulse placed at 7.5 ppm using a 22.7 ms sinc‐weighted Gaussian edit pulse. The water peak is assumed to be at 4.68 ppm. The differences between the edit‐on and edit‐off spectra determined GABA+ quantification.

### 2.5 1H‐MRS data processing

**Figure 1:**
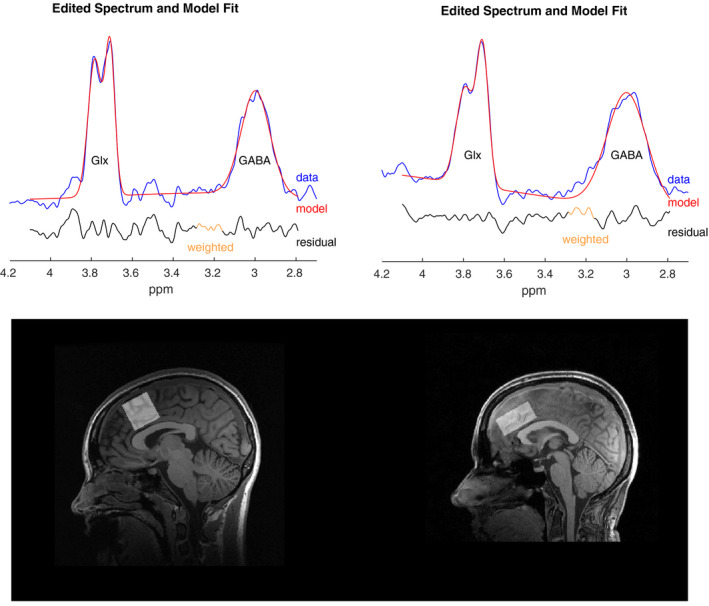
Upper panel (left HUH, right UNMH): The GABA‐spectrum in blue, the model fit is shown in red, while the residual is shown underneath, in black. Lower panel: Voxel placement at HUH (left) and UNMH (right). Both voxels are placed over the midline in the ACC. The voxel at HUH measures 30 × 30 × 30 mm giving a volume of 27 ml. The voxel at UNMH measures 40 × 26 × 20 mm, giving a volume of 20.8 ml

1H‐MRS processing of the UNMH and HUH data was completed through a common pipeline using Gannet (Edden, Puts, Harris, Barker, & Evans, 2014). This includes steps for combination of coil element data, time‐domain frequency and phase correction, exponential apodization, fast Fourier transform (FFT), pairwise rejection of data for which fitting parameters are >3 standard deviations from the mean and subtraction to generate the edited difference spectrum. Statistical Parametric Mapping 12 (SPM 12, https://www.fil.ion.ucl.ac.uk/spm/) completed segmentation of T1 images into gray matter, white matter, and CSF using a unified segmentation approach (Ashburner & Friston, 2005). Gannet performed GABA+ quantification including tissue‐class correction for gray matter, white matter, and CSF derived from the 3D T1 data (Edden et al., 2014; Harris, Puts, & Edden, 2015). SNR and linewidth estimates (full width at half maximum, FWHM) for HUH ranged from 12.0–30.7 and 13.1–29.5 Hz for GABA, 34,688–85,708 and 7.57–14.10 Hz for water, and 75.10–303.56 and 6.97–15.24 Hz for creatine. At UNMH, the corresponding ranges were 2.5–21.5 and 17.7–31.8 Hz for GABA, 127.20–1112.6 and 7.51–18.43 Hz for water, and 9.26–40.79 and 7.12–26.92 Hz for creatine. Preprocessed spectra and the corresponding model fit were inspected visually to check for aberrant features, and outlier rejection applied to flag suspicious estimates as described by Edden et al. (2014). Two subjects were removed from HUH. Spectra and model fits, as well as voxel placement for both sites, are shown in Figure 1. The choice of reference metabolite (creatine or water) has vendor and treatment‐related considerations. Siemens scanners over estimate water (Mikkelsen et al., 2019), but creatine (Cr) may change over the course of an ECT series (Njau et al., 2017). To balance vendor and treatment‐related changes, we use water (GABA+) as the reference for longitudinal comparisons, and creatine (GABA+/Cr) for cross‐sectional comparisons.

### 2.6 Statistical analysis

Statistical analysis was conducted using the R software version 3.4.0 (R Core Team, 2017). The Shapiro–Wilk test was used to test for normal distribution of the data. Summary statistics of sample characteristics were assessed using chi‐squared, two‐sample *t* tests, or nonparametric tests if data were not normally distributed. Results are reported as mean or median, ±*SD* or range, as specified in the text. The primary analyses focused on the combined data sets (HUH and UNMH, controlling for age, sex, and study site) with follow‐up tests for site‐specific data (controlling for age and sex). Repeated measures analysis of variance (RM‐ANOVA) assessed group differences (DEP and HC), the main effect of time, and group × time interaction. In addition, general linear models were constructed to assess the following: cross‐sectional differences in GABA+ between DEP and HC; longitudinal differences in GABA+ in DEP pre‐ and post‐ECT, changes in GABA+ related to change in MADRS or treatment number, and changes in GABA+ related to change in cognitive functioning (Trails A and B performance in seconds). Longitudinal change in GABA+ levels was measured as percentage change. The significance level for all tests was *p* < .05 with no multiple comparison correction.

## 3 RESULTS

### 3.1 Clinical outcomes and demographics

The joint analysis included 41 DEP and 35 HC. DEP and HC were not statistically different from each other based on age or sex. DEP improved in depression severity during the ECT series with 25 DEP meeting remission criteria and 16 DEP not meeting remission criteria. Details are given in Tables 1 and 2 shows the sample characteristics per site.

Twenty‐four subjects from both sites completed cognitive testing pre‐ and post‐ECT Trails A: median raw score in seconds before treatment = 35 (11–64), median raw score in seconds after treatment = 38.5 (15–90), *U* = 133.5, *p* = .65; Trails B: median raw score in seconds before treatment = 95 (55–360), median raw score in seconds after treatment = 107 (51–360), *U* = 128, *p* = .97. Both Trails A and B performance were normally distributed with all data within ± 3 standard deviations (*SD*).

**Table 1:** Sample characteristics shown as means (*SD*) for DEP versus HC

Variable	DEP	HC	*t* or *X^2^* (*p*‐value) for group differences
Number of subjects	41	35	
Age in years, mean (*SD*)	53 (16)	53 (14)	0.40 (.67)
Sex female/male	22/19	22/13	0.33(.56)
Pre‐ECT GABA+ level, mean (*SD*)	2.78 (0.37)	2.85 (0.58)	0.34 (.73)
Post‐ECT GABA + level, mean (*SD*)	2.76 (0.31)	2.94 (0.73)	0.90 (.37)
Delta GABA + level, mean (*SD*)	−0.02 (0.47)	0.09 (0.83)	−0.70 (.48)
Pre‐ECT MADRS, mean (*SD*)	32.40 (5.96)		
Post‐ECT MADRS, mean (*SD*)	11.17 (9.01)		
Delta MADRS, mean (*SD*)	21.23 (10.35)		
Pre‐ECT Trails A, median (range)	35 (11–64)		
Post‐ECT Trails A, median (range)	38.5 (15–90)		
Delta Trails A, mean (*SD*)	−1.38 (14.40)		
Pre‐ECT Trails B, median (range)	95 (55–360)		
Post‐ECT Trails B, median (range)	107 (51–360)		
Delta Trails B, mean (*SD*)	−1.04 (66.00)		

Note

For data which were not normally distributed median (range) is reported. Time for Trails is reported in seconds.

**Table 2:** Sample characteristics per site

Variable	HUH	UNMH	*t* or *χ* ^2^ (*p*‐value) for site differences
Number of DEP/HC	21/15	20/20	
Age in years, mean (*SD*) DEP/HC	44(19)/41(17)	63(10)/59(6)	−4.63(<.001)/−3.58(.002)
Sex: female DEP/HC	10/9	12/12	0.78 (.43)/−0.29 (.77)
HDRS pre‐ECT, mean (*SD*) DEP^a^		32.6 (7.6)	
Change HDRS, mean (*SD*) DEP		21.1 (14.0)	
MADRS score pre‐ECT, mean (*SD*)	34.7 (5.1)	30.0(5.9)	1.67 (.10)
Change in MADRS, mean (*SD*)	21.3(10.9)	21.2(10.0)	0.04 (.97)
Number of ECT treatments DEP, mean (range)	10.23 (3–18)	11.2 (6–18)	−0.79 (.45)
Pre‐ECT Trails A DEP, mean (*SD*)	33.85 (12.33)	38.72 (9.03)	−1.21 (.24)
Post‐ECT Trails A DEP, mean (*SD*)	38.33 (19.87)	42.33 (12.80)	−0.62 (.54)
Pre‐ECT Trails B DEP, mean (*SD*)	96.45 (27.82)	137.72 (94.24)	−2.18 (.04)
Post‐ECT Trails B DEP, mean (*SD*)	108.27 (51.06)	142.72 (82.62)	−1.39 (.18)
Antidepressant medications DEP^b^	13	19	
Lithium DEP	4	0	
Antipsychotic DEP	16	7	
Benzodiazepines DEP	0	6	
Subtype: melancholic		17/20	
Subtype: psychotic	1	10	

Note

Clinical and demographic characteristics of depressed subjects receiving ECT (DEP) and demographically matched healthy comparison subjects (HC) at Haukeland University Hospital (HUH) and University of New Mexico Hospital (UNMH).

^a^ HDRS‐24 raw scores from UNM site only. HDRS‐17 (subscale from HDRS‐24) was used for MADRS conversion equation.

^b^ Antidepressant medication includes the following: SSRI, SNRI, TCA, MAOi, Bupropion.

### 3.2 *Longitudinal differences in GABA*+ *between DEP and HC*

**Figure 2:**
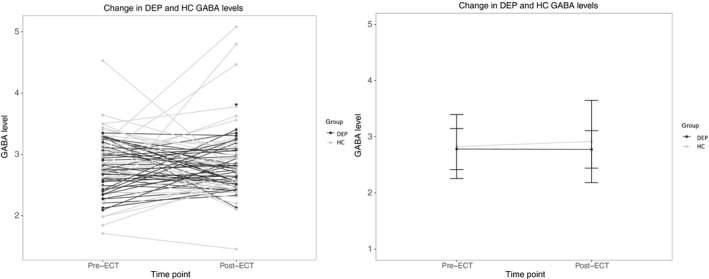
Left panel: Individual GABA levels for each DEP (black) and HC (gray) before and after ECT treatment. Right panel: Means of DEP and HC, before and after ECT, with their representative standard deviations

RM‐ANOVA revealed no significant effect of group (DEP, HC; *F* = 1.04, *p* = .31), time (*F* = 0.31, *p* = .58) or time × group interaction (*F* = 0.46, *p* = .50). Results are shown in Figure 2. For HUH, the time x group interaction was significant (*F* = 4.76, *p* = .03) but the main effects of group (DEP, HC; *F* = 1.14, *p* = .29) and time were not significant (*F* = 0.47, *p* = .50). For UNMH, group (DEP, HC; *F* = 7.10, *p* = .01) was significant, but time (*F* = 0.18, *p* = .67) and time × group interaction were not significant (*F* = 1.98, *p* = .17).

### 3.3 Cross‐sectional data: GABA+/Cr and Pre‐/post‐ECT DEP/HC contrasts

Linear models for the combined data did not reveal differences in GABA+/Cr levels between DEP and HC at pre‐ECT (combined: *t*_71_ = −0.36, *p* = .72; HUH: *t*_32_ = −1.15, *p* = .26; UNMH: *t*_36_ = 1.25, *p* = .22) or post‐ECT (combined: *t*_71_ = 1.17, *p* = .25; HUH: *t*_32_ = 1.11, *p* = .27; UNMH: *t*_36_ = 0.73, *p* = .47). The results for both sites combined were similar when using water as reference metabolite (GABA+) for the pre‐ (*t*_71_ = 0.34, *p* = .74) and post‐ECT contrasts (*t*_71_ = 0.91, *p* = .37).

### 3.4 Longitudinal analysis for DEP

Linear models did not reveal longitudinal changes in DEP GABA+ levels pre‐/post‐ECT (combined: *t*_36_ = 0.97, *p* = .34; HUH: *t*_17_ = 0.73, *p* = .47; or UNMH: *t*_16_ = 0.11, *p* = .91).

### 3.5 Remitters/nonremitters

Linear models did not reveal a difference in percentage change of GABA+ in remitters versus nonremitters (combined: *t*_36_ = 1.12, *p* = .27; HUH: *t*_17_ = 0.29, *p* = .78; or UNMH: *t*_16_ = −1.41, *p* = .18). There were no cross‐sectional differences between GABA+/Cr levels between remitters and nonremitters at baseline (combined: t36 = −0.14, *p* = .89; HUH: *t*_17_ = −0.73, *p* = .48; UNMH: *t*_16_ = 1.75, *p* = .10) or after treatment (combined: *t*_36_ = −1.16 *p* = .25; HUH: *t*_17_ = −0.97, *p* = .35; UNMH: *t*_16_ = −0.39, *p* = .70).

### 3.6 Relationship between GABA+, MADRS, and number of ECT treatments

Linear models did not demonstrate a relationship between change in MADRS and GABA+ when corrected for number of ECT treatments (combined: *t*_35_ = 1.12, *p* = .27; HUH: *t*_16_ = 1.92, *p* = .07; UNMH: *t*_15_ = −0.75, *p* = .47). Linear models did not demonstrate a relationship between number of treatments and GABA+ (combined: *t*_36_ = 0.05, *p* = .96; HUH: *t*_17_ = 0.64, *p* = .53; UNMH: *t*_16_ = −0.59, *p* = .56).

### 3.7 *Relationship between GABA*+ *and neuropsychological testing*

**Figure 3:**
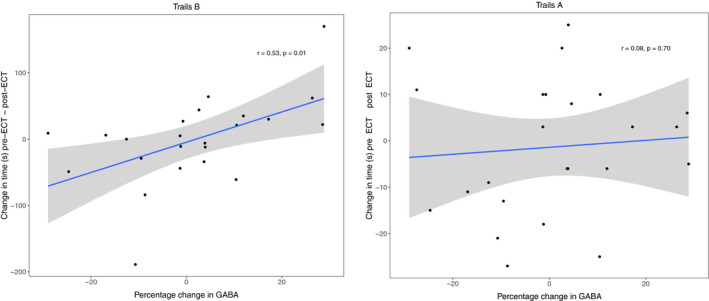
Association between percentage change in GABA+ levels and change in time solving neuropsychological tests. Left panel: Change in Trails A results (s) versus change in GABA+ levels; linear models (correcting for age, sex, and site) showed no significant relationship (*t*_19_ = 0.02, *p* = .98). The Pearson correlation is given in the figure. Right panel: Change in Trails B results (s) versus change in GABA+ levels; linear models (correcting for age, sex and site) demonstrated a significant relationship (*β* = 2.06, *t*_18_ = 2.4, *p* = .03). In both panels, the blue line represents the slope for the linear model with 95% confidence interval in gray. The Pearson correlation is given in the figure

Linear models did not demonstrate a relationship between change in performance on Trails A (low cognitive effort) and percentage change GABA + levels (combined: *t*_19_ = 0.02, *p* = .98; HUH: *t*_2_ = −1.46, *p* = .28; UNMH: *t*_14_ = 0.57, *p* = .58). Linear models demonstrated a relationship between change in performance on Trails B (high cognitive effort) and percentage change in GABA+ levels for both sites combined (*β* = 2.06, *t*_18_ = 2.4, *p* = .03) and UNMH (*t*_14_ = 2.67, *p* = .02), but HUH did not demonstrate this relationship (*t*_1_ = −6.20, *p* = .10). In the Trails B data, two subjects were possible outliers (+2.56 *SD* and–2.88 *SD* of the mean). Removing these subjects did impact the relationship between GABA+ and effortful cognitive performance (*β* = 1.2, *t*_16_ = 2.1, *p* = .06). The association between GABA+ levels and Trails B is illustrated in Figure 3, showing that increased change in effortful cognitive performance is associated with increased GABA+ levels after ECT.

## 4 DISCUSSION

Prior results from MRS studies looking at GABA+ in depressed subjects have been inconsistent. While several studies found reduced GABA+ in depressed subjects (Bhagwagar et al., 2007; Hasler et al., 2007; Sanacora et al., 2003) and positive change in GABA+ with medication treatment (Bhagwagar et al., 2004; Sanacora, Mason, Rothman, & Krystal, 2002), other studies contradict these findings (Godlewska et al., 2015; Hasler et al., 2005). We did not find a difference between GABA+ in DEP and HC, nor did we find a relationship between GABA+ and treatment response. Of the two 1H‐MRS studies looking at GABA+ in ECT, the positive study looking at occipital GABA+ pre‐ and post‐ECT (*n* = 8) was later contradicted by a slightly larger study (*n* = 11). Many of the studies mentioned above have small sample sizes. Studies with low sample size are more likely to overestimate effect size and have low reproducibility of results (Button et al., 2013). Also, previous studies focused on the occipital cortex, which has only been peripherally implicated in depression‐related neurocircuitry, whereas our study focused on the ACC, a brain region more directly implicated in depression (Hamilton et al., 2012). Pretranslational models with depression and electroconvulsive stimulations have also failed to demonstrate changes in hippocampal GABA+ (Biedermann et al., 2012). Our results argue against GABA+ in the brain being either a trait‐related biomarker of depression or a state‐related biomarker for a depressive episode.

A negative association between GABA+ and effortful cognitive performance as measured by the Trails B was found in subjects that completed cognitive testing. That is, from baseline to after treatment with ECT, as GABA+ increased, performance on Trails B worsened suggesting that increased GABA+ was associated with worsening of effortful cognitive functioning, such as, complex visual scanning, attention, and cognitive flexibility. This finding is consistent with prior research in adults with multiple sclerosis that found an association between greater levels of GABA+ in the posterior cingulate cortex and poor performance on the Trail Making Test (Cao et al., 2018). Several studies have linked baseline GABA+ and cognitive performance. Increased GABA+ in the frontal cortex was positively correlated with improved general cognitive performance as measured by the Montreal Cognitive Assessment (MoCA) (Porges et al., 2017), whereas decreased GABA+ in the dorsolateral prefrontal cortex (DLPFC) was associated with greater degradation of performance in trials with higher memory load as tested by a work memory paradigm created by the authors utilizing face cues (Yoon, Grandelis, & Maddock, 2016). Our results suggest that increased GABA+ specifically in the anterior cingulate was associated with worsening of effortful cognitive performance. GABA may be contextualized with other ECT‐cognitive biomarkers from structural MRI to help elucidate the mechanism of ECT‐mediated cognitive impairment (Laroy et al., 2019; Oostrom et al., 2018).

A strength of our investigation is the relatively large sample size, compared to prior studies. However, due to (a) the possibility of vendor‐specific GABA+ concentrations (Mikkelsen et al., 2019) and (b) a longer editing pulse used at UNMH compared with HUH (causing narrower band width and a resulting different amount of macromolecules contributing to the GABA+ signal) as well as other site differences, we have reported results separated by site in addition to our combined analysis. Our study has several limitations. First, while GABAergic medications (e.g. benzodiazepines) were avoided, subjects were continued on their psychotropic medications with minimal change throughout the ECT course, which could have affected GABA+ in the brain. Second, while our two independent sites had similar outcomes, they included different patient samples (age and diagnoses), anesthetics (methohexital at UNMH thiopental at HUH), and ECT stimulation parameters (pulse width, seizure titration vs. age‐, and demographic‐formulas). The site differences in ECT stimulation parameters, titration, and anesthetics also prohibited analyses focused on seizure duration to assess other aspects of the anticonvulsant hypothesis. Third, one of the technologic limitations of MRS is limited spatial resolution given the low concentrations of neurotransmitters when compared to water (Lee, Adany, & Choi, 2017). We can capture large volume changes in GABA+ using spectroscopy, but are unable to discern intravolume shifts of GABA+ (e.g., from intracellular spaces like the synaptic vesicle to extracellular spaces like the synaptic cleft). Fourth, the association between the change in GABA+ and worsening cognitive performance is a new finding, but cognitive testing was performed on a small subsample of patients at both sites and was limited to the Trail Making Test. This finding requires replication in studies with larger sample sizes with additional neuropsychological tests to assess the type and cognitive domain specificity of this relationship. For the method used in our analysis, effect sizes of ~15% should have been detectable for baseline difference of depressed subjects and healthy controls with group sizes of 20, for cross‐sectional data (Brix et al., 2017). Given the data of our study, a retrospective power analysis suggested that future studies would need *n* > 40 to have a power of 0.8 for detecting longitudinal GABA+ change in patients over ECT treatment.

Our results provide no support for changes in GABA+ as a mechanism of antidepressant action for ECT, but other lines of inquiry of the anticonvulsant hypothesis may warrant investigation. Although assessed cross‐sectionally with depressed subjects, ECT‐mediated changes in GABA+ from plasma or cerebral spinal fluid have yet to be investigated (Gerner & Hare, 1981; Kasa et al., 1982; Lu et al., 2014; Petty, Kramer, Dunnam, & Rush, 1990; Roy, Dejong, & Ferraro, 1991). Quantitative EEG assessments beyond seizure duration may also provide insights into anticonvulsant mechanisms (Coffey et al., 1995; Perera et al., 2004). The anticonvulsant hypothesis could be specific for a subtype of depression, and a more homogenous sample (e.g., melancholic depression) may demonstrate treatment‐responsive changes in GABA+. Alternatively, the focus on a single neurotransmitter or brain region may be insufficient to capture the complexity of the brain's adaptation to ECT. A pivot toward other theoretical frameworks such as neurotrophic (ECT promotes neurogenesis or synaptogenesis) or hyper‐connectivity (ECT modifies aberrant neural connectivity) to explain ECT’s mechanism of action for both clinical and neurocognitive outcomes may be warranted (Farzan, Boutros, Blumberger, & Daskalakis, 2014).

## 5 CONCLUSION

Our results provide no support for the anticonvulsant hypothesis as a mechanism of action for clinical outcome after ECT. We found no significant change between pre‐ and post‐ECT GABA+ in the anterior cingulate cortex relative to the demographically matched HC. Cross‐sectional analyses (pre‐ and post‐ECT) revealed no group differences in GABA+ between DEP and HC. Longitudinal analysis with DEP revealed no differences in GABA+. Finally, changes in GABA+ were unrelated to change in depression severity or number of ECT sessions. In contrast, our analysis suggests a negative effect of increased GABA+ during treatment on effortful cognitive performance as assessed by Trails B, showing that worsening effortful cognitive performance may be associated with increased GABA+ levels after ECT. Despite the insignificant findings with respect to depression outcomes, our research is important as it contributes to the limited evidence on this topic. Moreover, in contrast to previous studies, our study had a relatively large sample size (41 DEP) collected at two independent study sites with the same anatomic 1H‐MRS acquisition as well as neurocognitive tests (Trails Part A and Part B).

## CONFLICT OF INTEREST

None of the authors reported any conflicts of interest.

## AUTHOR CONTRIBUTION

LO, UK, JH, LE, CCA, and KJO have made contributions to conception and design. VJE, UK, LO, CCA, and TJ have contributed in the acquisition of data. VJE, JM, LE, ÅH, LO, CCA, TJ, JB, JP, VDC, and SMC have contributed in analysis and interpretation of data. All authors have been involved in drafting the manuscript or revising it critically for important intellectual content and have given final approval of the version to be published.

### Peer Review

The peer review history for this article is available at https://publons.com/publon/10.1002/brb3.1833.

## Data Availability

The data are not publicly available due to privacy or ethical restrictions.
