# Correction to “Anterior cingulate gamma‐aminobutyric acid concentrations and electroconvulsive therapy”

**DOI:** 10.1002/brb3.2835

**Published:** 2022-11-25

Erchinger, VJ, Miller, J, Jones, T, et al. Anterior cingulate gamma‐aminobutyric acid concentrations and electroconvulsive therapy. *Brain Behav*. 2020; 10:e01833. https://doi.org/10.1002/brb3.1833

In our paper, we wish to bring awareness to the following:

In Figure 3, the left and right panels were inadvertently swapped during production. Trails A should have appeared as the left panel and Trails B as the right panel. The corrected figure appears below.

**FIGURE 3:**
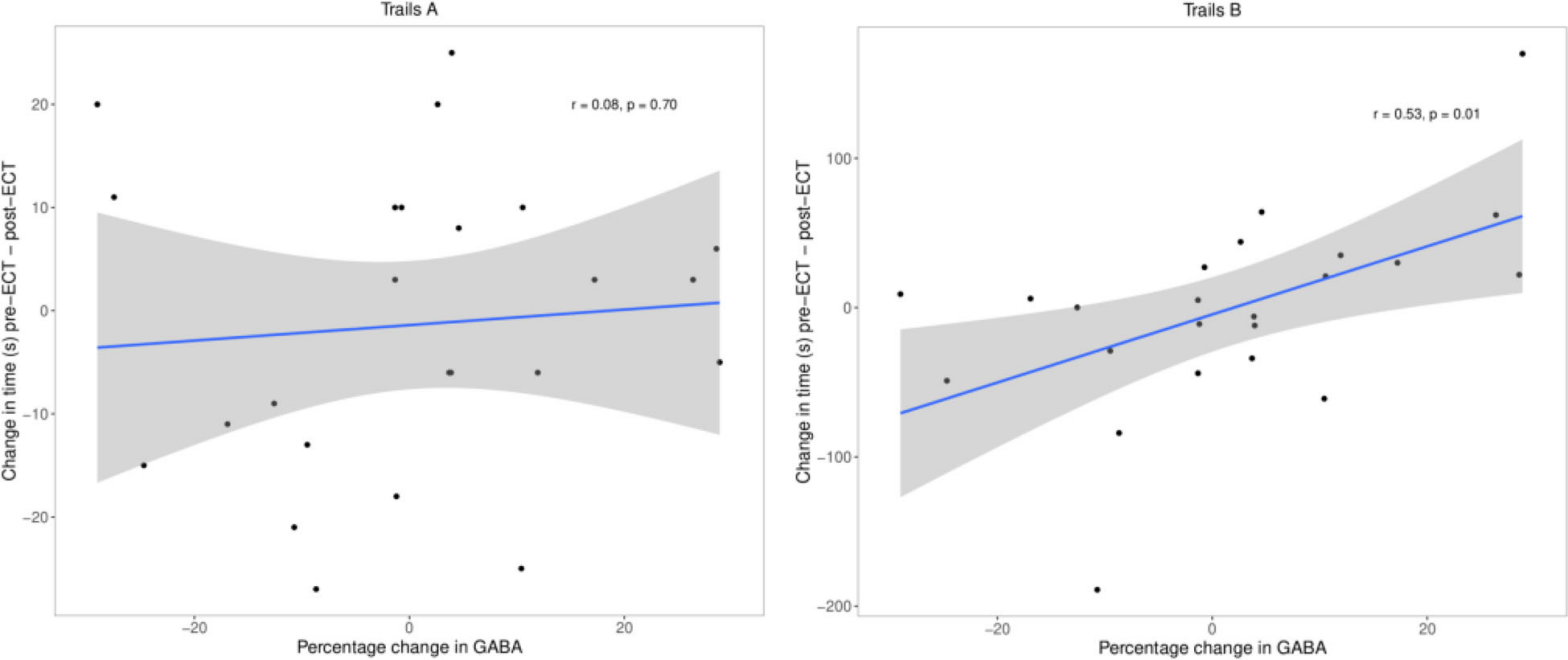
Association between percentage change in GABA+ levels and change in time solving neuropsychological tests. Left panel: Change in Trails A results (s) versus change in GABA+ levels; linear models (correcting for age, sex, and site) showed no significant relationship (*t*_19_ = 0.02, *p* = .98). The Pearson correlation is given in a figure. Right panel: Change in Trails B results (s) versus change in GABA+ levels; linear models (correcting for age, sex and site) demonstrated a significant relationship (*β* = 2.06, *t*_18_ = 2.4, *p* = .03). In both panels, the blue line represents the slope for the linear model with 95% confidence interval in gray. The Pearson correlation is given in the figure

Though the data is correct, there is an error in the interpretation of the finding in Figure 3 discussed in Sections 4 and 5 indicating a negative effect of increased GABA+ and that performance on Trails B worsened. The text should have stated that increased GABA+ is associated with improved performance on Trails B. The corrected version is found below.

## DISCUSSION

A positive association between GABA+ and effortful cognitive performance as measured by the Trails B was found in subjects that completed cognitive testing. That is, from baseline to after treatment with ECT, as GABA+ increased, performance on Trails B improved suggesting that increased GABA+ was associated with improvement of effortful cognitive functioning, such as, complex visual scanning, attention, and cognitive flexibility. This finding is inconsistent with prior research in adults with multiple sclerosis that found an association between greater levels of GABA+ in the posterior cingulate cortex and poor performance on the Trail Making Test (Cao et al., 2018). Several studies have linked baseline GABA+ and cognitive performance. Increased GABA+ in the frontal cortex was positively correlated with improved general cognitive performance as measured by the Montreal Cognitive Assessment (MoCA) (Porges et al., 2017), whereas decreased GABA+ in the dorsolateral prefrontal cortex (DLPFC) was associated with greater degradation of performance in trials with higher memory load as tested by a work memory paradigm created by the authors utilizing face cues (Yoon, Grandelis, & Maddock, 2016). Our results suggest that increased GABA+ specifically in the anterior cingulate was associated with improvement of effortful cognitive performance. GABA may be contextualized with other ECT‐cognitive biomarkers from structural MRI to help elucidate the mechanism of ECT‐mediated cognitive impairment (Laroy et al., 2019; Oostrom et al., 2018).

## CONCLUSION

In contrast, our analysis suggests a positive effect of increased GABA+ during treatment on effortful cognitive performance as assessed by Trails B, showing that improvement in effortful cognitive performance may be associated with increased GABA+ levels after ECT.

We apologize for the error in the original publication.

